# Structure Characterization and Antioxidant Activity of a Novel Polysaccharide from *Bacillus natto* Fermented Millet Bran

**DOI:** 10.3390/foods14020278

**Published:** 2025-01-16

**Authors:** Hanzhuo Zhang, Xia Fan, Wenjie Zhao, Fanqiang Meng, Fengxia Lu, Zhaoxin Lu, Haizhen Zhao

**Affiliations:** College of Food Science and Technology, Nanjing Agricultural University, Nanjing 210095, China; 2022108060@stu.njau.edu.cn (H.Z.); fanxia@njau.edu.cn (X.F.); 2022108078@stu.njau.edu.cn (W.Z.); mfq@njau.edu.cn (F.M.); lufengxia@njau.edu.cn (F.L.); fmb@njau.edu.cn (Z.L.)

**Keywords:** millet bran, *Bacillus natto*, polysaccharide, structural characterization, antioxidant activity

## Abstract

To improve the high-value application of millet bran, a water-soluble polysaccharide was extracted from fermented millet bran (FMBP) by using *Bacillus natto* fermentation. A neutral polysaccharide, FMBP-1, was separated and purified from FMBP using an anion exchange column. Its structure and antioxidant activity in vitro were characterized and determined. The molecular weight of FMBP-1 was 1.154 × 10^4^ Da, and its molecular weight distribution was relatively uniform. The monosaccharide composition, FT-IR, methylation, and NMR results indicated that FMBP-1 was only composed of glucose and was an α-(1→4)-D-glucan that branched at O-6 with a terminal 1-linked α-D-Glcp as a side chain. In addition, the antioxidant assays indicated that FMBP-1 possessed certain capacities for scavenging free radicals and reducing power, and this was in a concentration-dependent manner. This research will provide fundamental data regarding the structure–activity relationship of millet bran polysaccharides and provide a theoretical foundation for the high-value utilization of millet bran within the food and pharmaceutical industries.

## 1. Introduction

Millet is an ancient crop originating in China. Due to its characteristics of drought resistance, salt tolerance, and pest resistance, it has a wide distribution in China, India, Japan, North America, Australia, and Africa [1]. After millet is harvested, it needs to be processed before cooking and consumption to achieve good palatability. Namely, through the processes of hulling, milling, and polishing, refined millet is obtained. In this processing process, the by-product millet bran accounts for approximately 8% of the millet mass and is chiefly constituted of the seed coat, germ, and aleurone layer [2,3]. At present, there are relatively few functional products derived from millet bran, which generates meager economic benefits. Moreover, its taste and texture are subpar, so it is typically discarded or utilized as feed, leading to a very low utilization rate and added value [4]. However, fresh millet bran contains various beneficial compounds, such as proteins, dietary fiber, fats, vitamins, minerals, etc. These diversified phytochemical substances confer extensive physiological activities on millet bran, such as antioxidant capacities, immune regulation activities, and anti-tumor functions [5,6,7], which indicates that millet bran has great potential for development.

Polysaccharides are a type of high-molecular-weight carbohydrate with multiple functional characteristics and they are widely distributed in nature. As a natural active component, they can be applied in fields like medical pharmaceuticals, the food industry, agricultural antibiosis, and cosmetic additives, and possess the advantages of extensive sources, a high degree of safety, and good cell compatibility [8]. Similarly, the polysaccharide components in cereal bran also have various physiological functions that are beneficial to the human body, including antioxidant activity [9], lowering blood lipids [10], anti-tumor effects, and immune-regulating functions [11]. For a long time, people have overprocessed grain products to enhance their sensory quality, causing a considerable amount of polysaccharide bioactive components to flow into by-products like bran. To fully utilize these polysaccharides, researchers typically use various extraction methods and modification technologies to increase the yield of bran polysaccharides and obtain bioactive bran polysaccharides to enhance the added value of grains and maximize the utilization of resources. At present, the methods for extracting bran polysaccharides are diverse, mainly including ultrasonic extraction, microwave extraction, high-pressure pulse extraction, acid extraction, alkali extraction, and enzymatic hydrolysis [12]. These methods typically have problems such as chemical pollution, a low extraction rate, and a high cost [13], which have significant limitations in actual production and application. In addition, microbial fermentation is considered to be an economically viable and effective method and has attracted growing attention due to its advantages of mildness, environmental friendliness, selectivity, and high specificity [14]. Xu et al. [15] extracted polysaccharides from *Grifola frondosa*-fermented wheat grains, which presented significantly improved DPPH free radical scavenging abilities and immune-modulating effects. Wang et al. [16] found that polysaccharides from yeast-fermented *Lycium barbarum* had superior antioxidant and anti-aging effects compared to those from the traditional hot water extraction method. *B. subtilis natto* is a subspecies of *B. subtilis* that is widely used for its beneficial effects. During the fermentation process, it secretes various hydrolytic enzymes like protease, cellulase, nattokinase, and amylase, which can break down proteins, starch, and other substances in the substrate [17] and decompose cellulose to increase the content of soluble sugars [18]. Our previous study showed that soluble dietary fiber obtained from millet bran fermented by *B. natto* had a higher yield and better functional activity compared with that from unfermented millet bran [19,20]. However, determining which specific components play a role still requires further studies.

Therefore, this present study was designed to extract and purify polysaccharides from millet bran fermented by *B. natto*, and the structural and physicochemical properties of the purified polysaccharides were explored. Furthermore, their in vitro antioxidant activity was also assessed. These findings will contribute to clarifying the relationship between the structure and activity of millet bran polysaccharides and provide a theoretical foundation for their utilization and development in the food industry.

## 2. Materials and Methods

### 2.1. Materials and Chemicals

Millet bran was purchased from Xingtai city, Hebei province, China, defatted with n-hexane, and stored at −20 °C. *B. natto* was kept in the Enzyme Engineering Laboratory of the Department of Bioengineering, Nanjing Agricultural University (Jiangsu, China). Diethylaminoethyl (DEAE)-52 cellulose was obtained from Shanghai Yuanye BioTechnology Co., Ltd. (Shanghai, China). Monosaccharide standards were obtained from Sigma-Aldrich (St. Louis, MO, USA). All other chemicals and solvents were of analytically pure grade.

### 2.2. Extraction, Isolation, and Purification of Polysaccharides from Millet Bran

Following the method described by Yang et al. [20] with minor modifications, *B. natto* was inoculated into sterile nutrient broth (NB) medium, incubated at 37 °C, 180 r/min for 14 h, and the concentration of the bacterial solution was adjusted to 10^8^–10^9^ CFU/mL with sterile normal saline (0.9%, m/v). Then, 5% bacterial suspension was added to the sterilized millet bran medium (10 g of defatted millet bran, 0.5 g of sodium chloride, 0.1 g of glucose, and 100 mL of deionized water, adjusting the pH of the medium to 8.0, and sterilizing at 115 °C for 30 min) and cultivated at 37 °C, 180 r/min for 48 h. After that, the fermentation products were centrifuged. Four times the volume of 95% ethanol was added to the supernatant, and the resulting mixture was kept at 4 °C overnight. The crude polysaccharides precipitated by ethanol were collected through centrifugation and then freeze-dried. The proteins were removed by using the Sevag method [21], and then deproteinized fermented millet bran polysaccharide (FMBP) was obtained.

FMBP was fully dissolved in deionized water and centrifuged, and then the obtained supernatant was collected and filtrated through a 0.45 µm filter membrane and then loaded onto a DEAE-52 cellulose chromatography column. After overnight equilibrium, FMBP-1 was eluted with deionized water and different concentrations of NaCl. The content of polysaccharides in collection tube was measured by phenolsulfuric acid method [22], and the elution curve was plotted accordingly. Subsequently, the portions with high polysaccharide content in each fraction were combined, concentrated under vacuum, dialyzed (3500 Da), and freeze-dried. One major fraction, FMBP-1, was obtained for further analysis.

### 2.3. Structural Characterization

#### 2.3.1. Homogeneity and Molecular Weight Determination

The average molecular weight and polydispersity index (PDI) of FMBP-1 were determined using high-performance size-exclusion chromatography (HPSEC) combined with multi-angle laser light scattering (MALLS) detector (Wyatt Technology, Santa Barbara, CA, USA), differential refractive index (RI) detector (Agilent G7162A, Agilent, Santa Clara, CA, USA), and two connected columns, following the method of Gu et al. [23]. The sample was analyzed using 0.2 mol/L NaCl as the mobile phase at 1.0 mL/min. Data were processed using ASTRA 8 software.

#### 2.3.2. Monosaccharide Composition

Following the method of Peng et al. [24], FMBP-1 was dissolved in 2 M trifluoroacetic acid solution; then, it was hydrolyzed for 3 h at 120 °C. After the reaction was completed, the excess acid was dried via nitrogen blowing. Subsequently, methanol was used to wash and co-distill the obtained product. The hydrolyzed product was dissolved in deionized water and further analyzed by using a Dionex ICS-5000 system equipped with a CarboPAC PA20 (3 × 150 mm) anion exchange column. The column underwent elution with a mobile phase composed of A (H_2_O), B (0.1 M NaOH), and C (0.1 M NaOH-0.2 M NaAc) at 0.5 mL/min, with a 5 µL injection volume and a column temperature of 30 °C.

#### 2.3.3. Spectrometric Analysis

FMBP-1 was dissolved in deionized water, and then its UV spectrum was detected at 200–600 nm using a UV spectrophotometer (Shimadzu, Japan). KBr powder and FMBP-1 were mixed thoroughly, ground, and pressed into a uniform translucent ingot, and then the infrared spectrum of the sample (4000–500 cm^−1^) was detected using an infrared spectrometer (Nicolet NEXUS870, Thermo, Waltham, MA, USA).

#### 2.3.4. Triple Helix Structure Analysis

Equal volumes of 200 μmol/L Congo red and 2.5 mg/mL FMBP-1 solutions were mixed; then, NaOH was added to achieve final concentrations of 0, 0.1, 0.2, 0.3, 0.4, and 0.5 mol/L. After 10 min of reaction at 25 °C, the maximum absorption wavelength was measured.

#### 2.3.5. Methylation Detection

The methylation of FMBP-1 followed the method of Liu et al. [25]. Appropriate amount of FMBP-1 was dissolved in DMSO and reacted with anhydrous NaOH. Then, CH_3_I was slowly added into the tube for 1 h of methylation. The reaction was terminated with deionized water. After that, dichloromethane was added to extract the methylated product, which was further hydrolyzed with 2 M TFA. After hydrolysis, the products were reacted with 2 M NH_4_OH and 1 M NaBD_4_ for 150 min at 25 °C to achieve reduction and then acetylation with acetic anhydride and pyridine for 150 min at 100 °C. Finally, the acetylated products were extracted with dichloromethane, dried with anhydrous Na_2_SO_4_, and further analyzed by GC–MS system with an HP-5 capillary column (30 m × 0.32 mm × 0.25 μm). The temperature program for the HP-5 capillary column set was 140 °C (2 min hold) and then 3 °C/min to 230 °C (3 min hold) for a total runtime of 35 min. MS analysis was performed in a mass range of 50–350 *m*/*z* using high-purity helium as the carrier gas.

#### 2.3.6. Nuclear Magnetic Resonance (NMR) Spectroscopic Analysis

FMBP-1 (40 mg) was completely dissolved in 1 mL D_2_O and was transferred to an NMR tube for detection. At room temperature, NMR spectrometer (AVANCE NEO 500, Bruker, Fällanden, Switzerland) was used to record NMR spectra (^1^H, ^13^C, HSQC, COSY, and HMBC). The chemical shift was expressed in ppm.

#### 2.3.7. Scanning Electron Microscopy (SEM) Analysis

The FMBP-1 powder was placed on the sample stage and adhered with conductive tape. Then, under high vacuum conditions, gold powder was sputtered onto it using a sputter coater. At different magnifications (500× and 1000×), the sample’s morphological characteristics were observed with a scanning electron microscope (Sigma 300, Zeiss, Oberkochen, Germany).

#### 2.3.8. Particle Size Measurement

FMBP-1 was dissolved with deionized water to give a final concentration of 1.0 mg/mL. Particle size analysis was performed using a Zetasizer Nano ZS90 analyzer (Malvern Panalytical, Chipping Norton, NSW, Australia), and the detection was conducted at 25 °C with a scattering angle of 90° and a dispersant refractive index of 1.330.

#### 2.3.9. Thermal Stability Analysis

In the nitrogen atmosphere, TG analyzer (STA449F5, Netzsch, Munich, Germany) was used to perform the thermogravimetric analysis (TGA) of FMBP-1. FMBP-1 was placed in a platinum crucible and heated from 25 °C to 600 °C at 10 °C/min.

### 2.4. Determination of Antioxidant Activity In Vitro

The in vitro antioxidant capacity of FMBP-1 was evaluated by measuring the 2,2-diphenyl-1-picrylhydrazyl (DPPH), 2,2′-azinobis (3-ethylbenzthiazoline-6-sulfonic acid) (ABTS), hydroxyl radical scavenging ability, and reducing power. V_C_ was used as a positive control.

#### 2.4.1. DPPH Radical Scavenging Activity

The DPPH radical scavenging activity of polysaccharides was measured as described previously [26]. Equal volumes of 0.1 mM DPPH solution in ethanol and FMBP-1 at concentrations of 1–5 mg/mL were mixed. After incubating for 1 h in the dark, the absorbance at 517 nm was measured. The scavenging activity was calculated using the formula

Scavenging ability (%) = [1 − (*A*_S_ − *A*_C_)/*A*_0_] × 100 (1)

where *A*_S_ is the absorbance of the sample, *A*_C_ is the absorbance of anhydrous ethanol instead of DPPH, and *A*_0_ is the blank control absorbance of deionized water instead of FMBP-1.

#### 2.4.2. ABTS Radical Scavenging Activity

The ABTS radical scavenging activity of polysaccharides was measured as described in [21]. ABTS solution was prepared by mixing 7 mmol/L ABTS with 2.45 mmol/L potassium persulfate in equal volumes and incubating for 12 h in the dark. The solution’s absorbance was adjusted to 0.700 ± 0.020 at 734 nm using PBS. Amounts of 150 μL of ABTS working solution and 50 μL of sample at concentrations of 1–5 mg/mL were added to a 96-well plate, and absorbance at 734 nm was measured after 6 min in the dark. The scavenging activity was calculated as follows:

Scavenging ability (%) = [1 − (*A*_S_ − *A*_C_)/*A*_0_] × 100 (2)

where *A*_S_ is the sample absorbance, *A*_C_ is the absorbance of deionized water instead of the ABTS working solution, and *A*_0_ is the blank control absorbance (deionized water instead of FMBP-1).

#### 2.4.3. Hydroxyl Radical Scavenging Activity

The hydroxyl radical scavenging activity was measured as described in [27]. An amount of 2 mL of FMBP-1 solution (1–5 mg/mL) was mixed with 1 mL of 9 mM FeSO_4_, 1 mL of 9 mM salicylic acid-ethanol, and 1 mL of 8.8 mM H_2_O_2_. After incubation for 30 min at 37 °C, absorbance was measured at 510 nm. The scavenging activity was calculated as follows:

Scavenging ability (%) = [1 − (*A*_S_ − *A*_C_)/*A*_0_] × 100 (3)

where *A*_S_ is the sample absorbance, *A*_C_ is the absorbance of deionized water instead of H_2_O_2_, and *A*_0_ is the blank control absorbance with deionized water instead of the polysaccharide.

#### 2.4.4. Determination of Reducing Power

Following the method described by Jing et al. [28] with modifications, 2.5 mL of PBS and 1% potassium ferrocyanide solution were mixed with 2 mL of FMBP-1 solution (1–5 mg/mL) and incubated for 20 min at 50 °C. Then, 2.5 mL of 10% trichloroacetic acid was added, and the mixture was centrifuged. Next, 2.5 mL of the supernatant was mixed with 0.5 mL of 0.1% FeCl_3_ and 2.5 mL of deionized water. After 10 min of incubation at room temperature, absorbance was measured at 700 nm. A higher absorbance indicated greater reducing power.

### 2.5. Statistical Analysis

The experimental data were expressed as mean ± standard deviation of three replications and analyzed using GraphPad Prism 9.5.1 statistical software, and *p* < 0.05 was considered statistically significant.

## 3. Results and Discussion

### 3.1. Separation and Purification of FMBP-1

**Figure 1:**
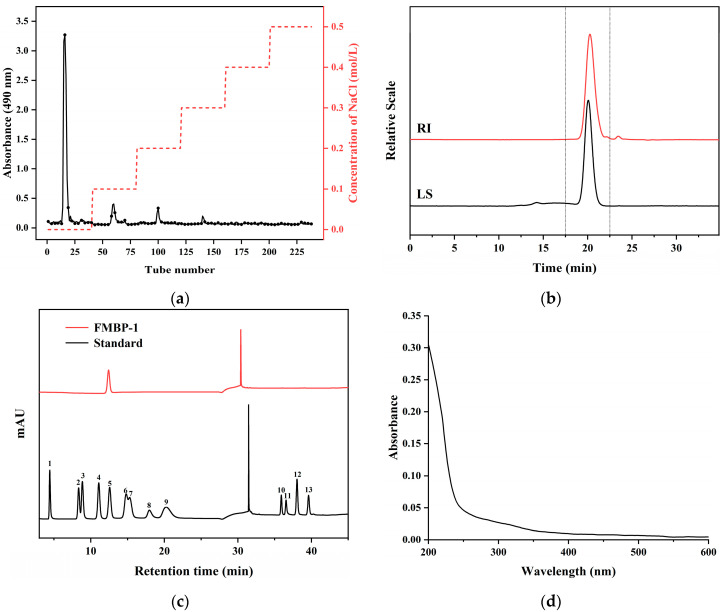
(**a**) Elution curve of FMBP on DEAE-52 cellulose column (with plotting performed on every third tube); (**b**) HPSEC-MALL-RI elution profile of FMBP-1; (**c**) ion chromatograms of standard monosaccharides and FMBP-1 (1-Fuc, 2-Rha, 3-Ara, 4-Gal, 5-Glc, 6-Xyl, 7-Man, 8-Fru, 9-Rib, 10-Gal-UA, 11-Gul-UA, 12-Glc-UA, 13-Man-UA); (**d**) UV spectrum of FMBP-1.

The crude polysaccharide FMBP was fractionated with a DEAE-52 cellulose column, and the resulting elution curve is presented in Figure 1a. Four fractions were obtained, and one main fraction named FMBP-1 (eluted with deionized water), which accounted for 31% of FMBP’s mass, was collected. Therefore, FMBP-1 was subsequently used for structural characterization and the activity assay.

### 3.2. Structural Characterization of FMBP-1

#### 3.2.1. Molecular Weight and Monosaccharide Composition Analysis

The HPSEC-MALLS-RI chromatogram of FMBP-1 showed a single symmetrical peak (Figure 1b), with consistent light scattering (LS) and refractive index (RI) signals, confirming its homogeneity [29]. FMBP-1 had a molecular weight of 11.54 kDa and a narrow polydispersity (PDI = 1.424).

The result of monosaccharide composition (Figure 1c) indicated that FMBP-1, composed of only glucose without uronic acid, was a neutral glucan.

#### 3.2.2. Analysis of UV Spectrum

Figure 1d presents the full-wavelength UV scanning spectrum of FMBP-1. No significant absorption peaks were observed at 260 nm or 280 nm, indicating that FMBP-1 contained minimal or no nucleic acids and proteins.

#### 3.2.3. FT-IR Analysis

The characteristics of molecular structure can be reflected in the intensity and position of the infrared absorption peaks, which could be used to determine the chemical groups of an unknown substance or detect the molecule’s structural composition [30].

**Figure 2:**
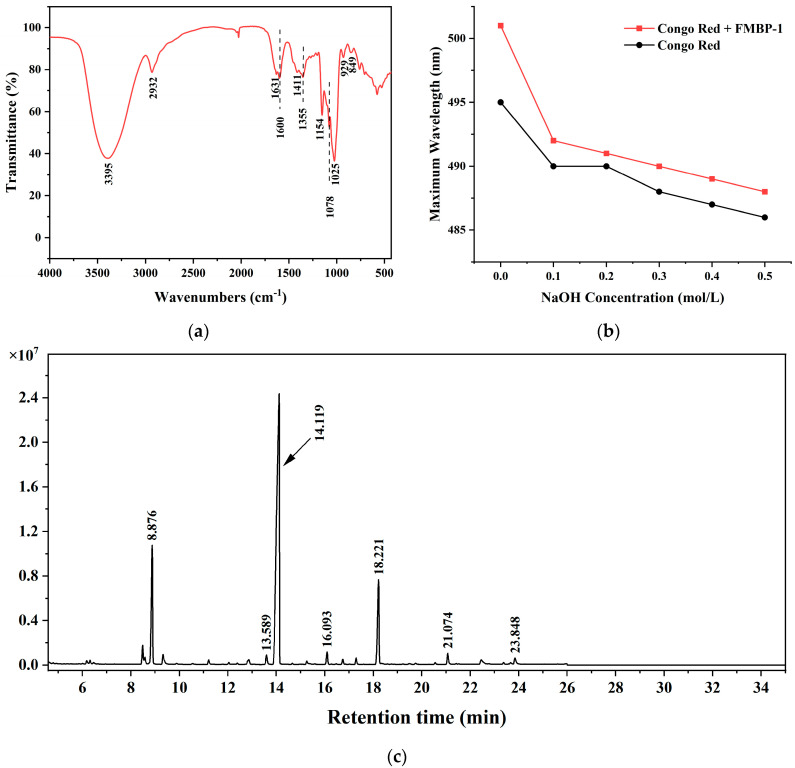
(**a**) FT-IR spectrum of FMBP-1; (**b**) maximum absorption wavelengths of FMBP-1 and Congo red mixed solution at different concentrations of NaOH; (**c**) GC chromatogram of alditol acetate derivatives from the methylated product of FMBP-1.

The infrared spectrum of FMBP-1 in the range of 4000–500 cm^−1^ is shown in Figure 2a. The strong and broad characteristic absorption peak at 3395 cm^−1^ corresponds to the stretching vibration of hydrogen bonds between or within the polysaccharide chains. Additionally, the absorption peak at 2932 cm^−1^ was attributed to the C-H stretching vibrations, both of which are characteristic features of polysaccharides [31]. There was no absorption peak at 1740 cm^−1^, which suggests that FMBP-1 does not have uronic acid. The absorption peaks at 1631 cm^−1^ and 1414 cm^−1^ correspond to C-O asymmetric stretching and C-H bending vibrations, respectively [32,33]. Meanwhile, the three absorption peaks at 1000–1200 cm^−1^ suggest that FMBP-1 contains pyranose rings [34,35]. The 829 and 849 cm^−1^ absorption peaks confirmed the existence of an α-structured glycosidic bond in FMBP-1 [31].

#### 3.2.4. Triple Helix Structural Analysis

Studies have shown that polysaccharides forming a complex with Congo red indicate triple helix structures, with a red shift in the maximum absorption wavelength as alkalinity increases [36]. In this present study, the maximum absorption wavelength of FMBP-1 and a Congo red mixed solution was determined at various NaOH concentrations. As seen in Figure 2b, with the increase in NaOH concentration, the maximum absorption wavelength of the mixed solution containing FMBP-1 and Congo red decreased gradually, while no red shift occurred, suggesting that FMBP-1 did not have a triple-helix structure.

#### 3.2.5. Methylation Analysis

The glucoside linkage can be effectively identified by methylation technology [37]. The mass spectrum of the methylated product (Figure 2c and Figure S1) was compared with that of the partially methylated alditol acetates [38]. As shown in Table 1, FMBP-1 contained three primary sugar linkages, 4-Glcp, t-Glcp, and 4, 6-Glcp residues in a 5:1:1 ratio, approximately, and two secondary sugar linkages: 3, 4-Glcp, and 6-Glcp residues. These results were consistent with the types of glycosidic linkages of cereal bran polysaccharides identified by Pasha and Ahmad using liquid chromatography–tandem mass spectrometry, where the linkages of 6-glucose, 4-glucose, and terminal glucose were prevalently present in bran samples [39].

#### 3.2.6. NMR Analysis

**Figure 3:**
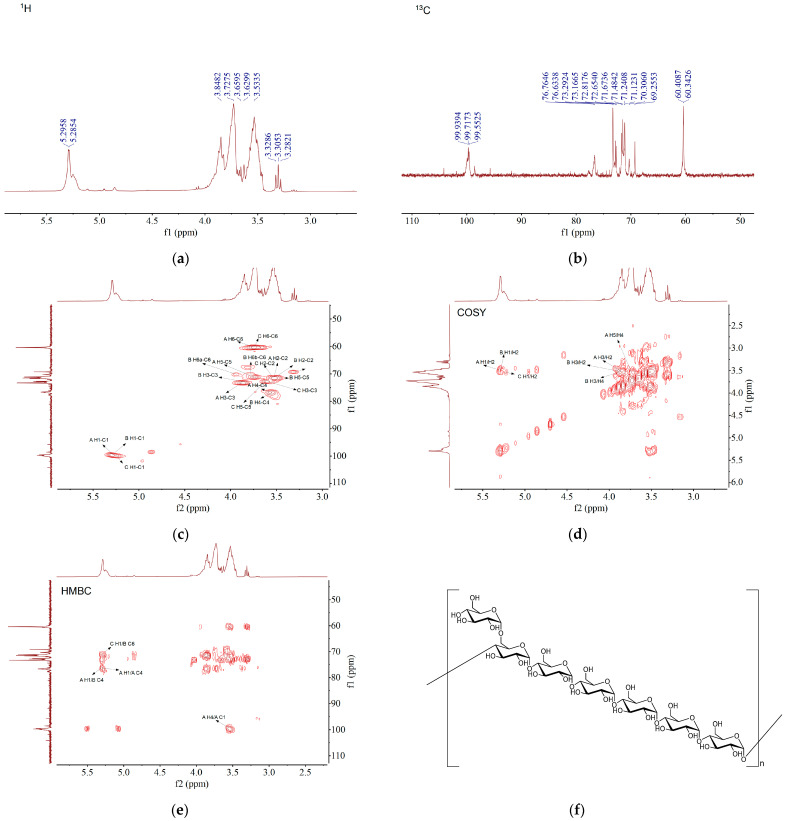
(**a**) ^1^H NMR; (**b**) ^13^C NMR; (**c**) HSQC NMR; (**d**) COSY NMR; (**e**) HMBC NMR; (**f**) predicted structure of FMBP-1.

It was found that there were three anomeric proton signals in the δ 4.5–δ 5.5 ppm regions at the ^1^H NMR spectrum of FMBP-1, namely, δ 5.29 ppm (residue A), δ 5.28 ppm (residue B), and δ 5.24 ppm (residue C), respectively, from Figure 3a. By combining this with the ^13^C NMR (Figure 3b) and the cross peaks of HSQC (Figure 3c), the anomeric carbon signals (δ 5.29/99.72, 5.29/99.55, and 5.24/99.94 ppm) of the residues were identified. The anomeric-related signals at other positions were relatively weak, confirming that they were residues with a lower content. These signals matched with the α conformation according to the literature, which showed that the ^1^H NMR signals in δ 4.9–5.5 ppm and δ 4.5–4.8 ppm were indicative of α and β configurations [40]. Combining the analysis of the methylation and the above study, the residues A, B, and C could be assigned to →4)-α-D-Glcp-(1→, →4, 6)-α-D-Glcp-(1→, and α-D-Glcp-(1→, respectively [41,42,43]. By combining this with the 2D NMR spectra, the carbon–hydrogen signal positions and structural correspondence information of the residues were successively assigned. The ^1^H and ^13^C NMR chemical shifts of FMBP-1 are shown in Table 2. A detailed analysis of residues A-C is presented below.

The H-1 and C-1 of residue A were at 5.29 and 99.72 ppm, respectively, which were typical terminal carbon proton signals of →4)-α-D-Glcp-(1→. In the COSY spectrum (Figure 3d), δ 5.29/3.52 and 3.52/3.86 ppm exhibited a COSY correlation, indicating that the signals of H-2 and H-3 were δ 3.52 and 3.86 ppm. In the HMBC spectrum (Figure 3e), H-3 (δ 3.86 ppm) was correlated with δ 76.63 ppm, inferring that δ 76.63 ppm was the C-4 signal. H-4 (δ 3.56 ppm) showed a COSY correlation with δ 3.74 ppm and an HMBC correlation with δ 60.41 ppm, suggesting that the signal at 3.74 ppm was attributed to H-5 and the signal at 60.41 ppm was attributed to C-6. Among them, C-4 (δ 76.63 ppm) had a downward shift in chemical shift value, and H-1 (δ 5.29 ppm) showed an HMBC correlation with C-4 (δ 76.63 ppm), indicating that the C-4 position formed a glycoside, and the residue A was determined to be →4)-α-D-Glcp-(1→ [41].

The H-1 and C-1 of residue B were at 5.28 and 99.55 ppm, respectively. In the COSY spectrum, the signals at δ 5.28/3.51, 3.51/3.85, and 3.85/3.55 ppm exhibited a COSY correlation, indicating that the chemical shift signal for H-2 was δ 3.51 ppm, H-3 was 3.85 ppm, and H-4 was 3.55 ppm, respectively. H-3 (δ 3.85 ppm) showed an HMBC correlation with δ 71.24 ppm, while δ 71.24 ppm also showed an HMBC correlation with δ 3.94 ppm, suggesting that the signals at δ 71.24 ppm and 3.94 ppm corresponded to C-5 and H-6, respectively. Additionally, C-4 (δ 76.76 ppm) presented a downward shift in chemical shift value, and H-1 (δ 5.29 ppm) showed an HMBC correlation with C-4 (δ 76.76 ppm), indicating the formation of a glycoside at the C-4 position. C-6 was at δ 70.31 ppm instead of the characteristic signal of unformed glycosides at δ 60–63 ppm, illustrating the formation of a glycoside at the C-6 position. Residue B was identified as →4, 6)-α-D-Glcp-(1→ [42].

For residue C, its H-1 was at δ 5.24 ppm, and the correlated C-1 was at δ 99.94 ppm. H-1 (δ 5.24 ppm) exhibited a COSY correlation with δ 3.55 ppm, indicating that δ 3.55 ppm was attributed to H-2. H-2 (δ 3.55 ppm) showed an HMBC correlation with δ 69.26 ppm, inferring that δ 69.26 ppm corresponded to the C-4 signal. H-4 (δ 3.31 ppm) showed an HMBC correlation with signals at 72.82, 72.65, and 60.34 ppm, which were attributed to C-3, C-5, and C-6, respectively [43].

Moreover, the methylation analysis of FMBP-1 showed that the content of →6)-Glcp-(1→ and →3, 4)-Glcp-(1→ was negligible, and there was basically no relevant signal in the NMR spectra, so they were not classified.

It could be speculated from the above analysis that FMBP-1 was an α-glucan with →4)-α-D-Glcp-(1→ as the main chain and had the branched α-D-Glcp-(1→ attached to →4, 6)-α-D-Glcp-(1→. Figure 3f presents the speculative repeating structural unit of FMBP-1. The structure of FMBP-1 was very similar to that of foxtail millet soluble dietary fiber extracted by Ren et al. [44] using amylase, protease, and cellulase. They had the same main chain and branch structure, and millet bran was a part of their foxtail millet. Therefore, we preliminarily speculated that amylase, cellulase, and glycoside hydrolase produced by *B. natto* played a role in the fermentation process. They used starch and insoluble dietary fiber or other sugars in millet bran as substrates and decomposed, modified, and even changed the configuration of glucoside bonds. Finally, the FMBP-1 with the above structure was isolated and purified.

#### 3.2.7. SEM Analysis

**Figure 4:**
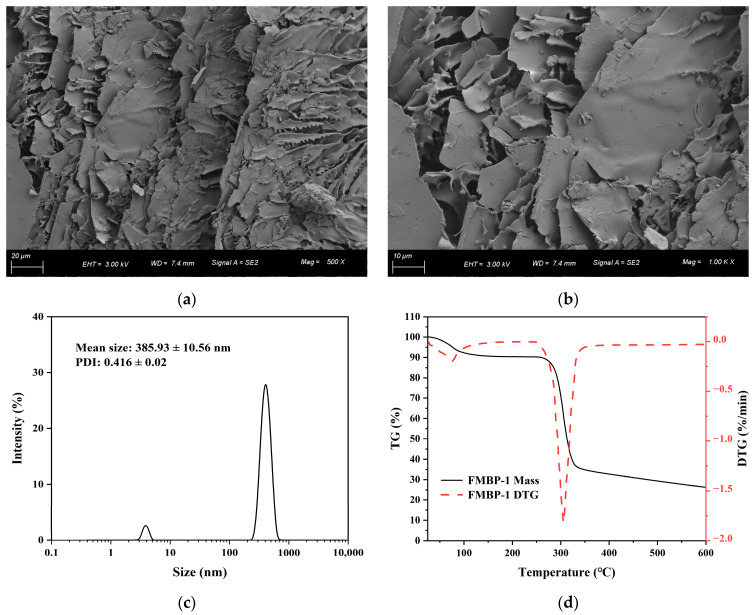
(**a**) SEM images of FMBP-1 at magnification of 500×; (**b**) SEM images of FMBP-1 at magnification of 1000×; (**c**) particle size distribution of FMBP-1; (**d**) thermogravimetry (TG) curve of FMBP-1.

Figure 4a,b shows that FMBP-1 presented a lamellar accumulation phenomenon. When magnified 500 times, the thickness of the lamellar structure was uneven, and there were protrusions of different stratifications on the surface. Enlarging the magnification revealed that the surface was relatively smooth and flat, with a small number of scattered fragments distributed around it. This phenotype might be caused by cross-linking between glycan chains, suggesting that the intermolecular forces were strong [45].

#### 3.2.8. Particle Size Analysis

The dispersion stability of the polysaccharide in the solution system could be reflected by particle size distribution, with a larger dispersion index indicating a wider distribution range of the sample [46]. Figure 4c showed that FMBP-1 was mainly concentrated at 300–500 nm in the solution, and the average particle size was 385.93 ± 10.56 nm. The PDI of 0.416 indicated that FMBP-1 had a relatively narrow distribution and had certain stability in the solution.

#### 3.2.9. Thermal Property Analysis

The thermal properties of polysaccharides are highly correlated with their structural and chemical characteristics [47]. Thermogravimetric analysis serves as a delicate technique for determining the thermal stability and decomposition temperature associated with a substance. The TG curve (Figure 4d) shows that there were three stages that occurred during the degradation of FMBP-1. The first stage was from 30 to 138 °C, with the decrease in weight during this stage resulting from the loss of bound water [8]. The second stage was from 258 to 330 °C, during which the weight loss was due to the disaggregation of FMBP-1 [48]. This involved the breakage of C-O and C-C bonds, their cracking into volatile components such as CO_2_ and H_2_O, and eventually forming a polynuclear aromatic and graphite–carbon structure. The weight loss rate was the highest in this stage at 52.8%. The third stage was from 330 °C to 600 °C. The degradation rate was relatively slow and tended to be stable at the end, and the final residue was 26.3% of the initial mass. The above results indicated that FMBP-1 exhibited good thermal stability.

### 3.3. Antioxidant Activity Analysis of FMBP-1 In Vitro

Cells can produce free radicals during normal metabolic processes, and the formation and removal of free radicals exist in a balanced state. Once this balance is disrupted, excess free radicals will lead to oxidative stress in cells, thus causing serious damage to tissues [49]. Studies have shown that the antioxidant effect of polysaccharides is usually due to hydrogen atom transfer (HAT) and single electron transfer (SET), that is, by providing hydrogen atoms to neutralize free radicals and by transferring an electron to neutralize free radicals and metal ions to delay or reverse oxidative stress damage [50].

**Figure 5:**
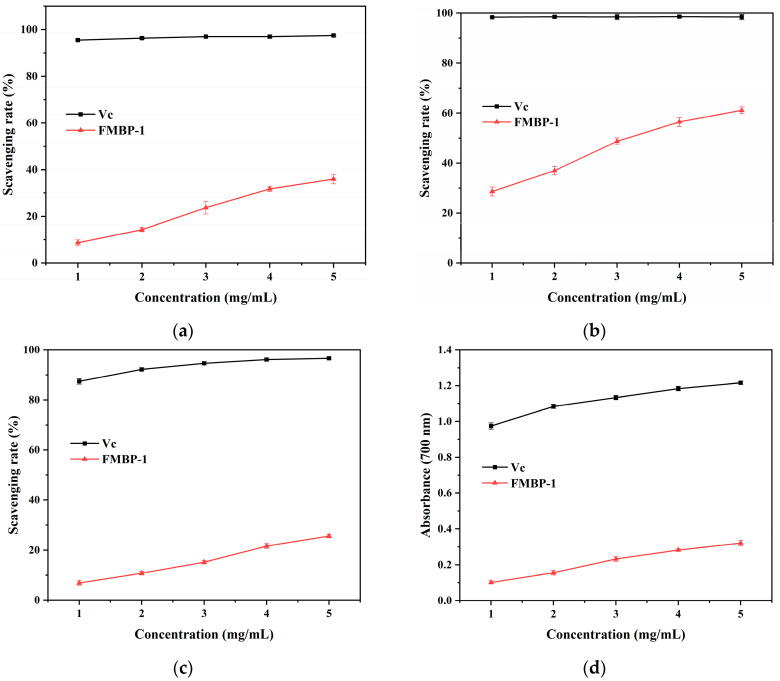
(**a**) Scavenging activity of DPPH radical; (**b**) ABTS radical; (**c**) hydroxyl radical; (**d**) reducing power ability.

As shown in Figure 5, the antioxidant activities of FMBP-1 became stronger as the polysaccharide concentration increased, showing a concentration-dependent relationship within the tested dosage range (1.0–5.0 mg/mL). The free radical scavenging rate and reducing ability of FMBP-1 were considerably lower than those of the positive control Vc (*p* < 0.05). When the sample concentration was 5 mg/mL, its DPPH, ABTS, hydroxyl free radical scavenging rate, and reducing capacity were 35.99%, 61.13%, 25.67%, and 0.32 (OD_700nm_), respectively. The different antioxidant levels measured by the four methods might have been due to the different ability of FMBP-1 as a hydrogen donor to provide hydrogen atoms and electrons to bind different free radicals, and the HAT and SET mechanisms might have acted in different proportions [50]. Overall, FMBP-1 had moderate antioxidant capacity. The structural characteristics of polysaccharides often affect their antioxidant activities. Previous studies have shown that polysaccharides with low molecular weights had better antioxidant activity since there were more reducing hydroxyl end groups to neutralize and scavenge free radicals under the same unit mass [51]. Polysaccharides composed of a greater variety of monosaccharides had better antioxidant activity than those composed of a single monosaccharide. Moreover, the higher the content of uronic acid in polysaccharides, the stronger the antioxidant capacity, as the charged groups in this structure had higher availability [52]. Chen et al. [53] showed in their review that the main glycosidic bond connection of antioxidant polysaccharides contains α-1,4-D-Glc, and the (1→4) bond is a flexible structural unit, which is easier to dissolve in solution and gives easier access to free radicals. In summary, FMBP-1 had a relatively small molecular weight and had α-1,4-D-Glc as its main chain, but it was a neutral polysaccharide containing only glucose. The different effects of these structural characteristics on the antioxidant aspect led to the moderate antioxidant activity of FMBP-1. FMBP-1 may act as an antioxidant to neutralize free radicals to avoid tissue damage. The antioxidant activities of FMBP-1 were comparable to those of other α-glucan, such as *Morchella sextelata* polysaccharide (MSP1-1) [54], *Mirabilis himalaica* (Edgew) heim polysaccharide (MHHP) [55], and *Gastrodia elata* Blume polysaccharide (GEP2-6) [56], which had an α, 1-4 main chain. Among them, MSP1-1 exhibited significantly lower ferric-reducing ability than that of Vc, and its DPPH free radical scavenging rate was 43.41% at 4 mg/mL. MHHP showed a relatively low antioxidant capacity with a maximum hydroxyl radical scavenging capacity of 18.63% at 10 mg/mL. GEP2-6 also presented weak Trolox equivalent antioxidant capacity. It can be seen from the above that α-glucans with an α-1, 4 main chain usually had modest antioxidant activity. In addition, it is evident that there was a strong correlation between the structure and activity of polysaccharides. The analysis of the polysaccharide structure was helpful for rapidly determining their possible biological activity.

In addition to antioxidant activity, α-1,4-glucan-neutral ginger polysaccharide [38] and wheat bran polysaccharide [57] with a similar structure to FMBP-1 had immunomodulatory activity, which could promote the proliferation of Raw264.7 cells, stimulate the production of immune factors, and activate the pathways of NF-κB and MAPK. Based on the characteristic that structure determines function, FMBP-1 may have potential immunoregulatory activity. It is necessary to conduct in-depth research on the immunomodulatory activity of FMBP-1 in the future.

## 4. Conclusions

In this study, a homogeneous α-glucan FMBP-1 composed only of glucose was obtained from *B. natto* fermented millet bran. The molecular weight of FMBP-1 was 11.54 kDa, and it consisted of repeating units with a main chain of →4)-α-D-Glcp-(1→ and a branch chain of α-D-Glcp-(1→ connected to →4,6)-α-D-Glcp-(1→. FMBP-1 presented good dispersion stability and thermal stability, while it had no triple-helix structure. Moreover, the antioxidant capacity of FMBP-1 was dose-dependent. The results elucidated the structural types of fermented millet bran polysaccharides and showed that FMBP-1 might be used as a potential bioactive substance. The following work will focus on the immunoregulatory activity of FMBP-1 and adequately test its digestion and safety before it is applied to the food industry in the future.

## Figures and Tables

**Table 1:** Methylation analysis of FMBP-1.

Linkage Type	Methylated Sugar	Retention Time (min)	Mass Fragments (*m*/*z*)	Molar Ratio (%)
t-Glcp	2,3,4,6-Me_4_-Glcp	8.876	87, 102, 118, 129, 145, 161, 162, 205	14.88
6-Glcp	2,3,4-Me_3_-Glcp	13.598	87, 99, 102, 118, 129, 162, 189, 233	1.32
4-Glcp	2,3,6-Me_3_-Glcp	14.119	87, 102, 113, 118, 129, 162, 233	70.21
3,4-Glcp	2,6-Me_2_-Glcp	16.093	87, 118, 129, 143, 185, 203, 305	1.32
4,6-Glcp	2,3-Me_2_-Glcp	18.221	85, 102, 118, 127, 159, 162, 201, 261	12.28

**Table 2:** ^1^H NMR and ^13^C NMR chemical shifts (ppm) of FMBP-1 in D_2_O.

Code	Glycosyl Residues	Chemical Shifts (ppm)					
		H1/C1	H2/C2	H3/C3	H4/C4	H5/C5	H6/C6
A	→4)-α-D-Glcp-(1→	5.29	3.52	3.86	3.56	3.74	3.75–3.65
		99.72	71.48	73.29	76.63	71.12	60.41
B	→4,6)-α-D-Glcp-(1→	5.28	3.51	3.85	3.55	3.45	3.94, 3.80
		99.55	71.67	73.16	76.76	71.24	70.31
C	α-D-Glcp-(1→	5.24	3.55	3.60	3.31	3.60	3.75–3.65
		99.94	71.67	72.82	69.26	72.65	60.34

## Data Availability

The original contributions presented in this study are included in the article/Supplementary Material. Further inquiries can be directed to the corresponding author.

## Supplementary Materials

The following supporting information can be downloaded at: https://www.mdpi.com/article/10.3390/foods14020278/s1, Figure S1: GC–MS profiles of partially methylated alditol acetates of FMBP-1.
