# Supplementary material for: Structure Characterization and Antioxidant Activity of a Novel Polysaccharide from Bacillus natto Fermented Millet Bran
Source: Foods. 2025 Jan 16;14(2):278. doi: 10.3390/foods14020278 (PMC11765371; doi:10.3390/foods14020278)
Supplement: Supplementary file 1 [file foods-14-00278-s001.zip › foods-3362148-supplementary.pdf]

Supplementary material

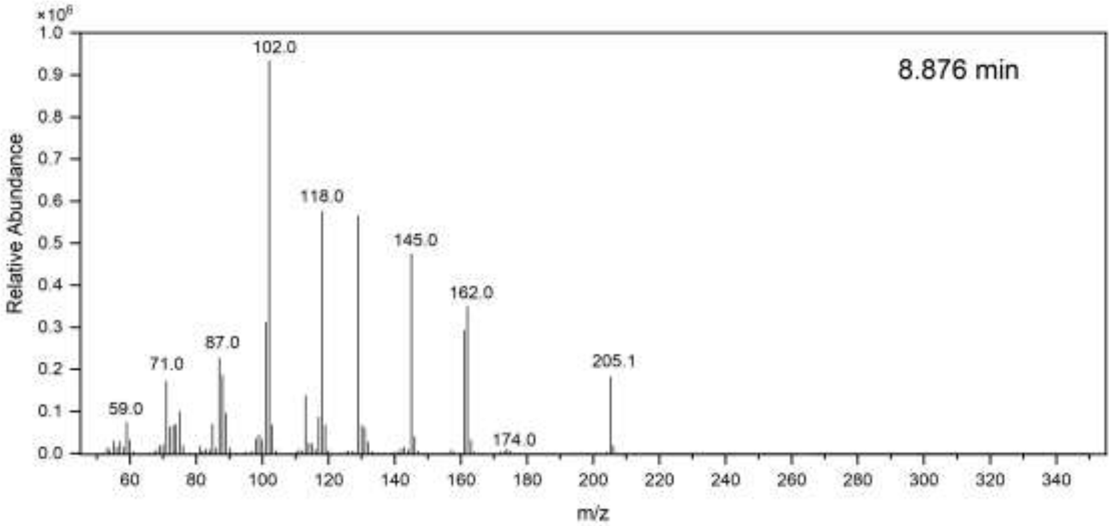

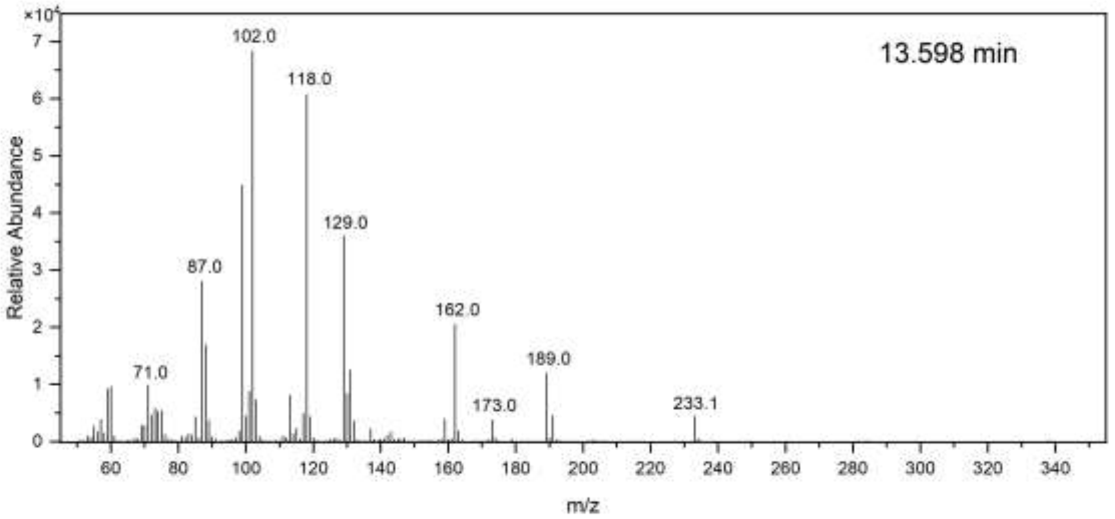

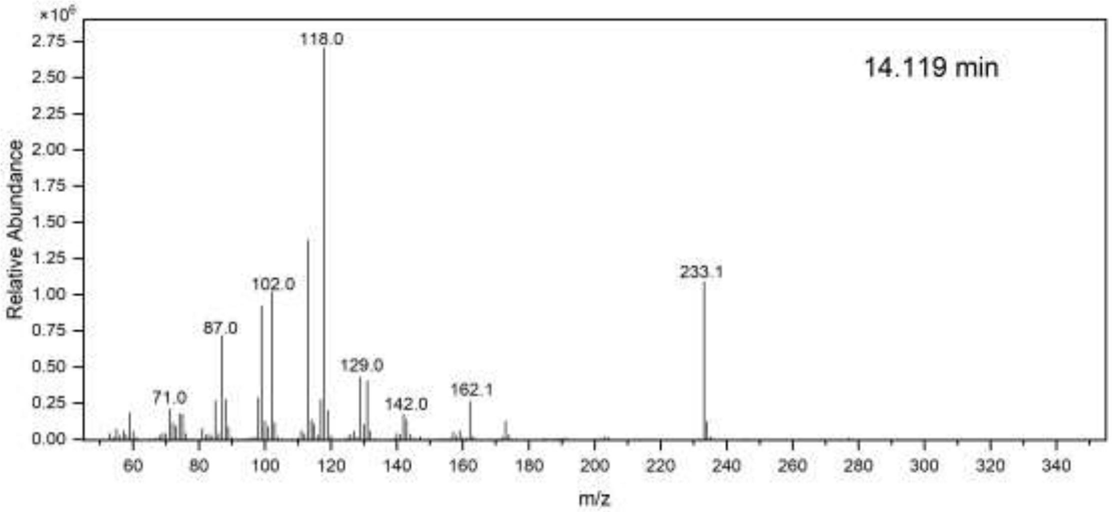

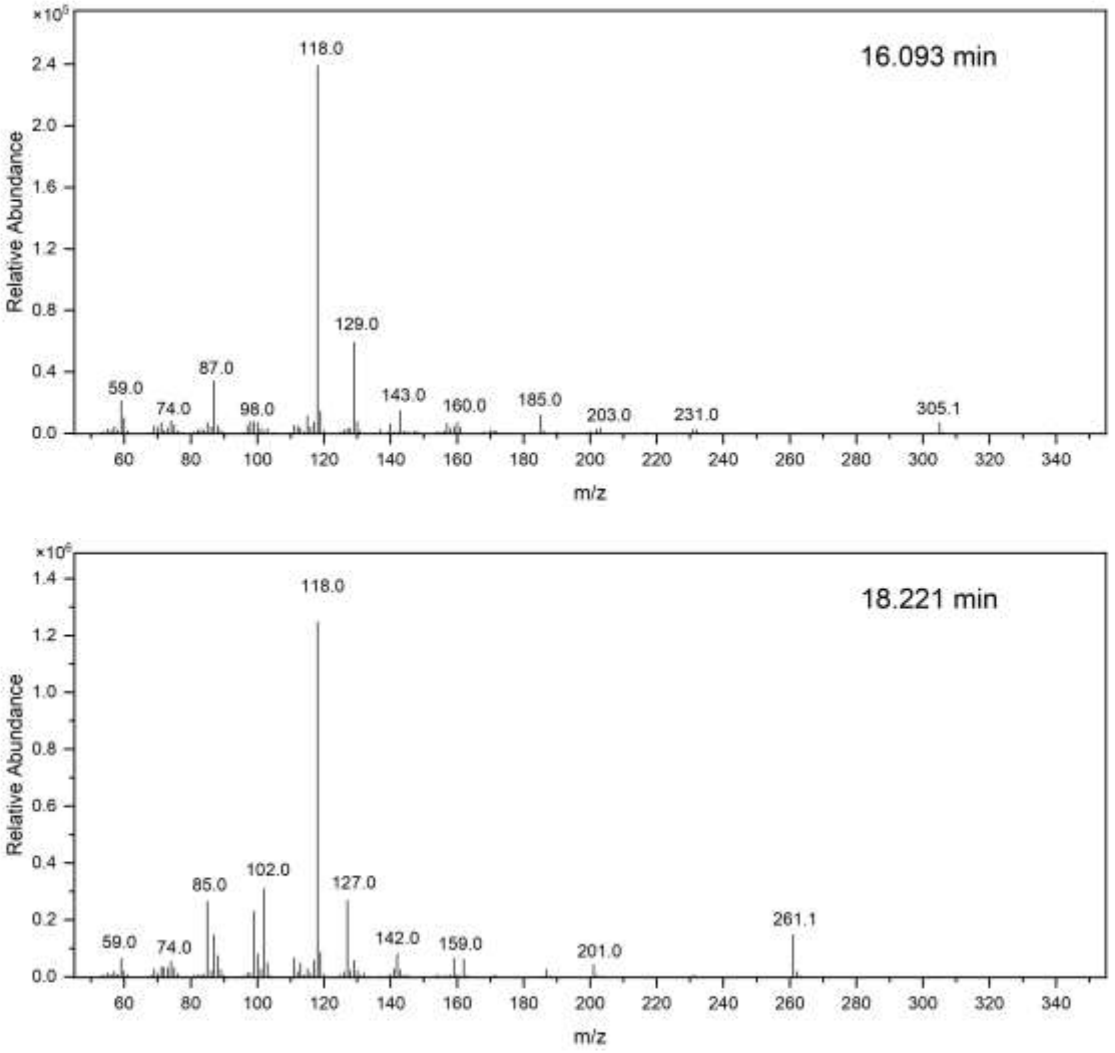

Figure S1. GC–MS profiles of partially methylated alditol acetates of FMBP-1
